# Prenatal exposure to legacy contaminants and visual acuity in Canadian infants: a maternal-infant research on environmental chemicals study (MIREC-ID)

**DOI:** 10.1186/s12940-020-0567-2

**Published:** 2020-02-07

**Authors:** C. Polevoy, T. E. Arbuckle, Y. Oulhote, B. P. Lanphear, K. A. Cockell, G. Muckle, D. Saint-Amour

**Affiliations:** 1 Centre de recherche, CHU Sainte-Justine, QC, Montréal, Canada; 2 Département de psychologie, Université du Québec à Montréal, QC, Montréal, Canada; 3 Département d’ophtalmologie, Université de Montréal, QC, Montréal, Canada; 4 Population Studies Division, Environmental Health Science and Research Bureau, Health Canada, Ottawa, ON Canada; 5 Department of Biostatistics and Epidemiology, School of Public health and health Sciences, University of Massachusetts at Amherst, Amherst, MA USA; 6 BC Children’s Hospital, Simon Fraser University, Burnaby, Canada; 7 Nutrition Research Division, Food Directorate, Health Products and Food Branch, Health Canada, Ottawa, ON Canada; 8 École de psychologie, Université Laval and Centre de recherche du CHU de Québec-Université Laval, Québec, QC Canada

**Keywords:** Visual acuity, Infants, Heavy metals, Persistent organic pollutants, Background levels, MIREC study

## Abstract

**Background:**

Prenatal exposure to environmental contaminants can have deleterious effects on child development. While psychomotor, cognitive and behavioural outcomes have been investigated in relation to chronic exposure, the associations with visual functions remains unclear. The present study’s aim was to assess the associations of prenatal exposure to legacy persistent organic pollutants and heavy metals with visual acuity in Canadian infants. The potential protective effects of selenium against mercury toxicity were also examined.

**Methods:**

Participants (mean corrected age = 6.6 months) were part of the Maternal-Infant Research on Environmental Chemicals (MIREC) study. Concentrations of polychlorinated biphenyls (PCBs), polybrominated diphenyl ethers (PBDEs), lead and mercury were measured in maternal blood during pregnancy, as well as in the cord blood. The Teller acuity card test (TAC) (*n* = 429) and the visual evoked potentials in a sub-group (*n* = 63) were used to estimate behavioural and electrophysiological visual acuity, respectively. Multivariable linear regression models were used to investigate the relationship between exposure to each contaminant and visual acuity measures, while controlling for potential confounders. Breastmilk selenium, which was available for about half of the TAC and VEP samples, was also taken into account in the mercury models as exploratory analyses.

**Results:**

We observed no significant associations between exposure to any contaminants and TAC. Analyses revealed a negative trend (*p* values < 0.1) between cord blood lead and mercury and electrophysiological visual acuity, whereas PCB and PBDE showed no association. When adding breastmilk selenium concentration to the mercury models, this association became statistically significant for cord concentrations (β = − 3.41, 95% CI = − 5.96,-0.86), but also for blood levels at 1st and 3rd trimesters of pregnancy (β = − 3.29, 95% CI = − 5.69,-0.88). However, further regression models suggested that this change in estimates might not be due to adjustment for selenium, but instead to a change in the study sample.

**Conclusions:**

Our results suggest that subtle, but detectable alterations of infant electrophysiological visual acuity can be identified in a population prenatally exposed to low mercury concentrations. Compared to behavioural visual acuity testing, electrophysiological assessment may more sensitive in detecting visual neurotoxicity in relation with prenatal exposure to mercury.

## Background

The impact of prenatal exposure to legacy environmental contaminants (ECs) in humans, such as polychlorinated biphenyls (PCBs) or methylmercury, have been widely investigated. Fetuses and young children are recognized as the most vulnerable population to the effects of contaminant exposure because they have increased absorption rates relative to body weight, and their immature body systems are not yet prepared to effectively metabolize, detoxify and excrete toxicants [1]. Most chemicals can reach the fetus through transplacental transfer [2], while breastfeeding also constitutes a significant exposure source in infants, especially for persistent lipophilic compounds [3]. Exposure to some ECs are known to interfere with gene expression and development of the central nervous system, possibly leading to potential neurodevelopmental effects later in life [4].

Heavy metal exposure is ubiquitous in human populations. Although government policies have resulted in reductions of lead (Pb) in many commercial products over the past few decades (e.g., gasoline, paint, food cans), which substantially decreased the blood lead levels in the general population, exposure still occurs through dust inhalation, food and water ingestion [5]. Mercury (Hg), converted into its organic and most toxic form, methylmercury, accumulates and biomagnifies in the food chain. The main source of methylmercury exposure is from consumption of certain species of fish, and Hg is also present in air and water because of human activities [6]. Prenatal exposure to Hg following poisoning incidents has been linked to severe and various neurological impairments [7], and a large number of studies have subsequently shown alterations of cognitive functions, including attention, language, motor and intellectual performance in children exposed to chronic lower levels of Pb or Hg [8–10]. Despite an accumulating body of epidemiological studies that reported that acute and long-term exposure to heavy metals alters the integrity of the visual system (e.g., colour discrimination, contrast sensitivity, visual field constriction) [7, 11, 12], few studies have assessed the impact of chronic lower level exposure on visual function. Of those, studies conducted among fish-eating populations in the Faroe Islands, Madeira (Portugal) or Northern Québec (Nunavik) have shown that in school-aged children prenatal exposure to elevated levels of Pb or Hg was associated with visual alterations, as measured using visual evoked potentials (VEPs), [13–16]. These epidemiological studies are in line with the laboratory data demonstrating that exposure to several chemicals in animal models, in particular Hg and Pb, cause cellular alterations in the visual pathway but also in the eye, including the retina [17].

Among the persistent organic pollutants (POPs), polychlorinated biphenyls (PCBs) are one of the most studied organochlorine compounds in relation to human neurotoxicity. PCBs were commercialized in the early 1930s and primarily used in the industrial and commercial fields (e.g., caulking compounds, lubricants, transformers, adhesives), until their production was banned in the United States and in Canada by the end of the 1970s [18]. Although their concentrations have tended to decrease over time [19], they are still measurable in biological samples of the general population. Structurally and chemically, polybrominated diphenyl ethers (PBDEs) are similar to PCBs. PBDEs were mainly used as flame retardants in electronic and various other consumer products (e.g., home appliances, computers, furniture); however, manufacturing with PBDEs was stopped in 2008 in Canada [20]. The general population is exposed to PBDEs via diet, but most significantly through house dust, so that young children are 3- to 9-fold more exposed than adults [21]. High doses of prenatal PCBs following poisoning incidents have been linked to severe developmental impairments, either neurological, sensorial or motor [22], while the effects of this type of exposure are not documented for PBDEs. Chronic lower levels of PCBs and PBDEs have also been associated with alterations in various cognitive functions (e.g., global intellectual functioning, attention and executive functions, memory) [23, 24]. Regarding visual functions, chronic prenatal exposure to PCBs has been linked to VEP alterations in children [16]. No information on the effects of PBDEs on visual functions was available in the literature.

As noted above, while cognitive outcomes have been investigated in relation to prenatal exposure to environmental chemicals, associations with visual development have been understudied. Although some alterations of visual processing have been reported, few studies have specifically examined visual functions, such as visual acuity, and even less among low-level exposed populations. To our knowledge, only two studies have examined the effects of prenatal background exposure to ECs (i.e., Pb, organic solvents and organophosphate insecticides) and visual acuity during development, showing subtle but measurable deleterious effects [25, 26]. Given the potentially asymptomatic nature of the visual alterations in the general population, visual acuity testing is entirely appropriate since it is objective, effective and unbiased. It also offers advantageous methodological factors, supporting its use as a potential marker of developmental effects among infants: no verbal responses are needed, it is easy to administer, score, analyze and interpret, and standardized norms based on age are available [27]. Given that visual acuity development begins during the prenatal period and matures until school age, it offers a large window of vulnerability to the effect of toxic insults.

The goal of the present study was twofold. The primary aim was to assess the associations between prenatal low-level exposure to two classes of ECs, i.e., POPs (PCBs and PBDEs) and heavy metals (Hg and Pb), and visual acuity development both behaviourally and electrophysiologically in infants. Considering neuroprotective properties of Selenium (Se) in relation with Hg [28], the secondary aim was to explore the potential influence of selenium on the associations between Hg exposure and the visual outcomes.

## Methods

### Study setting and population

The participants were recruited as part of *Maternal-Infant Research on Environmental Chemicals* (MIREC), which is a Canadian national-level multisite pregnancy cohort and infant follow-up study. Additional information about the MIREC cohort is detailed elsewhere [29]. Briefly, a cohort of 2001 pregnant women was recruited from prenatal clinics during their first trimester (6 to < 14 weeks) during a 4-year enrolment period (2008–2011). Of those, 525 mother-infant pairs participated in a follow-up infant development study (MIREC-ID), which aimed to assess the role of prenatal exposure to ECs on infant health around 6 months of age, including visual acuity. Criteria for inclusion in the MIREC-ID study included birth as singleton, at ≥28 weeks of gestation, and without major congenital birth defects or neurological disorders. For the present study, out of the 525 mother-infant pairs, ninety infants (17.1%) did not complete the visual assessment (e.g., lack of time or cooperation from the participant), and 6 (1.4%) were not included in the study because of suspected ocular abnormality (e.g., congenital cataracts, retinoblastoma) as screened by the red reflex test [30], leaving a final sample of 429 mother-infant pairs. Considering that prenatal exposure to ECs might possibly be related to prematurity [31] and/or low birth weight [32], analyses were conducted among the sample as a whole. Sensitivity analyses were also conducted to examine the potential influence of premature and/or low birth weight infants (see *Statistical Analysis*). Age at testing time was adjusted to gestational age for premature babies by subtracting the number of weeks of prematurity from chronological age at testing time (corrected age).

The MIREC and MIREC-ID studies were reviewed and approved by the ethics committees at Health Canada and recruitment sites. Parents consented prior to participation and were provided with information on the design and objectives of the study. All tests and measures were non-invasive and conducted by trained research nurses or research professionals.

### Data collection

#### Biospecimen collection and chemical analyses

Prenatal POPs concentrations (PCBs and PBDEs) were measured during the first trimester (6–13 weeks) in the maternal blood plasma and at birth in the cord blood plasma [33]. However, POPs cord blood concentrations were excluded from analysis because 70.4–79.6% and 78.4–79.3% of the values were below the limit of detection (LOD), for PCBs and PBDEs congeners, respectively. Maternal blood was collected in 10 mL K2 EDTA tubes; plasma was transferred into 2.5 mL pre-cleaned glass vials (Supelco®) and stored at − 20 °C. POPs concentrations were measured using Agilent 6890 Network or 7890A gas chromatograph coupled to an Agilent 5973 Network or 5975C mass spectrometer (Agilent Technologies; Mississauga, Ontario, Canada). Among all POPs congeners available in the MIREC study (i.e., 28, 52, 74, 99, 101, 105, 118, 138, 146, 153, 156, 167, 170, 180, 187 for PCBs, and 28, 47, 99, 100, 153 for PBDEs), the sum of congeners − 118, − 138, − 153 and − 180 (∑PCBs) was used as an indicator of PCB exposure, and the sum of congeners − 47, − 99 and − 153 (∑PBDEs) for PBDE exposure. This summation metric has been used for PCBs and PBDEs in epidemiological studies [34, 35], considering that these congeners are highly correlated, generally found in higher levels in human blood samples and therefore, detectable in most participants. Total plasma lipid concentrations were also measured. Total cholesterol (TC), free cholesterol (FC), triglycerides (TG) and phospholipids (PL) levels were measured in the samples by enzymatic methods combined with colorimetry (in g/L) at the laboratory of Centre Hospitalier de l’Université Laval (CHUL; Québec, QC, Canada) and were used to calculate the total lipid level as 1.677*(TC-FC) + FC + TG + PL [36].

Lead and mercury were measured in maternal whole blood collected during the first (6–13 weeks) and third (32–34 weeks) trimester visits and in cord blood at delivery [37]. Samples were analyzed by sample dilution followed by inductively coupled plasma mass spectrometry (PerkinElmer ELAN ICP-MS DRC II) analysis [ICP-MS DRC-II; Elan Perkin Elmer]. For analysis, prenatal exposure to heavy metals was estimated from two measures: 1) the cord whole blood concentrations, and 2) the average of maternal whole blood concentrations taken during the first and third trimesters, which were highly correlated (*r*_*s*_ = > 0.70, *ps* < 0.001) (see Table 4). This average measure of both exposure time points was obtained for 94.2% of the mothers, whereas for the remaining 5.8%, only one trimester was used because the other one was missing (4.9 and 1.0% had only 1st or 3rd trimester data, respectively). Cord blood measures were obtained for 100% of the participants. All maternal and cord blood chemical analysis were carried out at the Laboratoire de Toxicologie, Institut National de Santé Publique du Québec (INSPQ) (Québec, QC, Canada), accredited by the Standards Council of Canada under ISO 17025 and CAN-P-43.

Selenium was measured in breast milk, which has been shown to be strongly correlated with selenium concentrations in cord blood and in maternal plasma during gestation (*r*_*s*_ > 0.6) [38, 39]. Milk samples were expressed by the participants over multiple days between the 2nd and 10th weeks post-delivery. The milk was collected in 16 oz. wide mouth amber I-CHEM® glass jars with fluoropolymer resin-liner polypropylene closure (Thermo Fisher Scientific, Rockwood, TN, USA) and 16 oz. wide mouth TraceClean® clear plastic polyethylene jars (VWR International, Radnor, PA, USA). Milk samples were retained in the participant’s refrigerators (~ 4 °C) for up to three days, or if collected over longer periods were stored in their freezers (~ − 20 °C) at home until sample collection was complete. Samples were shipped frozen to Quebec Region Food Laboratory of Health Canada, an ISO 17025 accredited program, and analyzed for selenium concentration. After microwave digestion in a mixture of nitric and perchloric acids, samples were analyzed on an Agilent 7500c ICP-MS with Micromist nebulizer. Quality assurance was provided through contemporaneous analysis of standard materials including NIST 1549 (Non-fat Milk Powder). The limit of detection by this method was 0.004 μg/g for 1 g sample.

### Visual acuity assessments

Two methods were used for assessing visual acuity: A behavioral method that measures subjectively the child’s behavior in responses to visual stimuli (gratings) presented on cards (Teller Acuity Cards, TAC), and an electrophysiological method that measures objectively the child’s brain in responses to visual stimuli (gratings) presented on a computer screen (Visual Evoked Potentials, VEP). In both cases, grating visual acuity is expressed as the number of cycle per degree of visual angle (cpd) that are seen, where lower cpd score means worse acuity. A higher visual acuity score is expected using VEP compared to TAC, due to multiple physiological and methodological factors, which are explained in details elsewhere [40]. The correlation between the visual acuity scores obtained from both methods in infants is typically modest. In the present study, the Pearson coefficient between TAC and VEP was 0.2, which justify the use of both methods for assessing the impact of prenatal exposure to environmental contaminants on the visual function.

### Teller acuity cards

The Teller Acuity Cards™ (TAC) are recognized internationally as a rapid, reliable and effective assessment tool to assess subjective (behavioural) visual acuity development in infants [41, 42]. The TAC was administered at various Canadian sites of the MIREC study for a total of 429 valid scores. Of note, 17 subjects (3.96%) showed extreme low scores based on the 99% confidence interval test norms. The TAC test is based on an automatic behaviour response called preferential looking, which is a preference to look at a stimulus versus a plain area when both are presented at the same time [43]. The TAC uses laminated cards (25.5 × 55.5 cm), containing on one side the stimulus (a 12 × 12 cm square wave grating, with a contrast of 60–70%) and a gray area on the other side. The visual acuity score is expressed in cycles per degree of visual angle (cpd), where a higher value indicates better visual acuity. Trained research nurses, masked to the exposure concentrations, administered the TAC for the duration of approximately 10 min, under binocular viewing conditions. Each infant was seated on their parent’s lap at 55 cm from the cards, which were presented progressively, from wide to narrower gratings (from 1.3 to 38 cpd). Visual acuity was estimated as the finest or thinnest stimulus that elicited a visual preference (expressed behaviourally by the infant), as judged by the experimenter looking through a small peephole in the middle of the card. A more detailed description of the TAC procedure is offered elsewhere [40].

### Visual evoked potentials

In addition to the TAC test, a subsample of infants (*n* = 72), only at the Montreal site of the MIREC study (CHU Sainte-Justine), also completed an “electrophysiological” visual acuity assessment using the sweep VEP paradigm [44]. Nine participants (12.5%) were excluded because of incomplete VEP data due to tiredness or lack of cooperation, leaving a final sample of 63 participants. Infants were seated on their parent’s lap at 85 cm from the stimuli presented on a computer CRT monitor, while electrophysiological activity was recorded at the occipital cortex (Oz) using Ag/AgCl active electrodes. Stimuli were generated by Presentation software® and consisted of vertical sinusoidal gratings with a spatial frequency ranging from 1.0 to 13.5 cpd, with 80% contrast. Stimuli were swept at 12 reversals/s, with each grating displayed and recorded in 1-s segments. The protocol was repeated approximately 5 times depending on the infant’s cooperation. EEG data was acquired using the V-Amp system (Brain Products, Inc., Munich, Germany) and VEP signals were recorded and analyzed using Analyzer® software. The software calculated the mean amplitude value for each grating and estimated background noise levels using neighboured frequencies. The MATLAB® program (MathWorks, Inc.), was used to estimate visual acuity thresholds by applying linear extrapolation of the amplitude as a function of the spatial frequency. A more detailed procedure and characteristics of the extrapolation method can be found elsewhere [40].

### Statistical analysis

The distributions and frequencies of all variables and covariates of interest were first examined for normality. To satisfy these assumptions, a log-10 transformation was applied to all exposure data (∑PCBs, ∑PBDEs, Hg and Pb). Moreover, a square root transformation was applied to the TAC scores, whereas VEP scores were normally distributed. After transformations, all data were normally distributed (skewedness values between ±2). A semi-parametric left-censored method was applied to model the concentrations below the LOD for our ECs of interest. Specifically, we used a regression on order statistics method [45], which performs regression on data greater than the LOD, assuming log normal percentiles to predict concentrations ≤ LOD. This procedure has been shown to be robust under the log-normal distribution [46].

Separate linear regression analyses were conducted to investigate the associations between each contaminant (∑PCBs, ∑PBDEs, Hg and Pb) and visual outcomes (TAC and VEP) as categorical or continuous variables. Using the first approach, EC exposures were divided categorically based on the sample size of the outcome, which was into two groups for VEP scores (≤median and > median, or “low” and “high” exposure level), and into three groups for TAC scores (0-33rd, 33-66th, 66-100th percentile ranks or “low”, “moderate” and “high” exposure). In both cases, the lowest exposure group was treated as the reference group. We next generated a multiple linear regression model on the continuous data (i.e., exposure was treated continuously) to specifically test linearity and improve statistical power to our models.

#### Potential confounders

Based on prior knowledge and the literature, the following infant-related potential confounders were examined: gestational age at delivery, sex (male vs. female), weight and length at testing time, corrected age at testing, breastfeeding duration (< 3, 3- < 6, ≥6 months) and head circumference. Maternal-related variables, obtained from medical records and questionnaires administered during each trimester and at delivery, were also examined: age, education (less than undergraduate university degree vs. undergraduate university degree or more), race/ethnicity (white/Caucasian vs. other), marital status (married or with same partner > 1 year vs. other), household income (<$10,000–50,000, 50,001–100,000, > 100,000), birth country (foreign vs. Canada), pre-pregnancy BMI (< 25, 25–29.9, ≥30 kg/m^2^), number of previous viable pregnancies or parity (0, 1, ≥ 2), smoking status during pregnancy (never, former or quit during 1st trimester vs. current or quit during the 3rd trimester), any alcohol use during the first trimester of pregnancy (yes vs. no), and total lipids level.

Age and sex of the infant were systematically entered in the regression models. Final covariates were selected based on theoretical a priori and statistical associations. As such all variables associated with both prenatal concentrations (independent variables) and the visual outcomes (dependent variables) at *p* ≤ 0.2 were considered as potential confounding factors and included in the final regression model. Thus the common covariable set applied to each model was the following: infant corrected age at testing time and infant sex, breastfeeding duration, maternal education, maternal birth country, maternal alcohol consumption during pregnancy. One variable was retained as a risk factor (i.e., smoking status during pregnancy) due to its association at *p* ≤ 0.2 only with the visual outcomes and was included in the final set of covariates. Total lipids in maternal blood plasma was treated as an additional covariate in our POPs models to control for their bioaccumulative properties [47]. Finally, despite the role of Se against Hg neurotoxicity in humans is not well established [48], we also examined selenium as a potential confounder or effect modifier when investigating Hg associations.

Three specific sensitivity analyses were performed; 1) by excluding the 17 participants with outlier scores on the TAC, 2) by excluding low birth weight and/or premature babies, and 3) by including the variable Site in the models, as the participants of the current study were recruited and followed from 7 sites across Canadian cities (Vancouver, Hamilton, Kingston, Ottawa, Montreal, Halifax). These analyses were conducted only on the TAC models due to the small sample size for VEP. Moreover, additional analyses were carried out to investigate the potential influence of selenium on Hg models; 1) by adding selenium as a covariate to the Hg regression models, for both TAC and VEP models and 2) by using a stratified analysis based on selenium concentration (< or ≥ 19.80 ng/g), only for the TAC model.

All statistical analyses were performed using SPSS version 23 (IBM Corp.). The censoring method used to impute data with exposures <LOD was executed from the package NADA (*Nondetects and Data Analysis for Environmental Data*) of the R libraries.

## Results

### Descriptive statistics

Maternal characteristics are shown for the current study sample (*n* = 429) and for the entire MIREC cohort (*n* = 1983) in Table 1. In the current study, average maternal age at enrollment was 31.9 years, and women were well-educated, mostly born in Canada (86.7%) and married or with some partner for one year or more (95.1%). The majority did not smoke (94.4%) or drink (83.7%) during pregnancy. Overall, these characteristics are quite similar to those observed for the entire cohort.

**Table 1:**
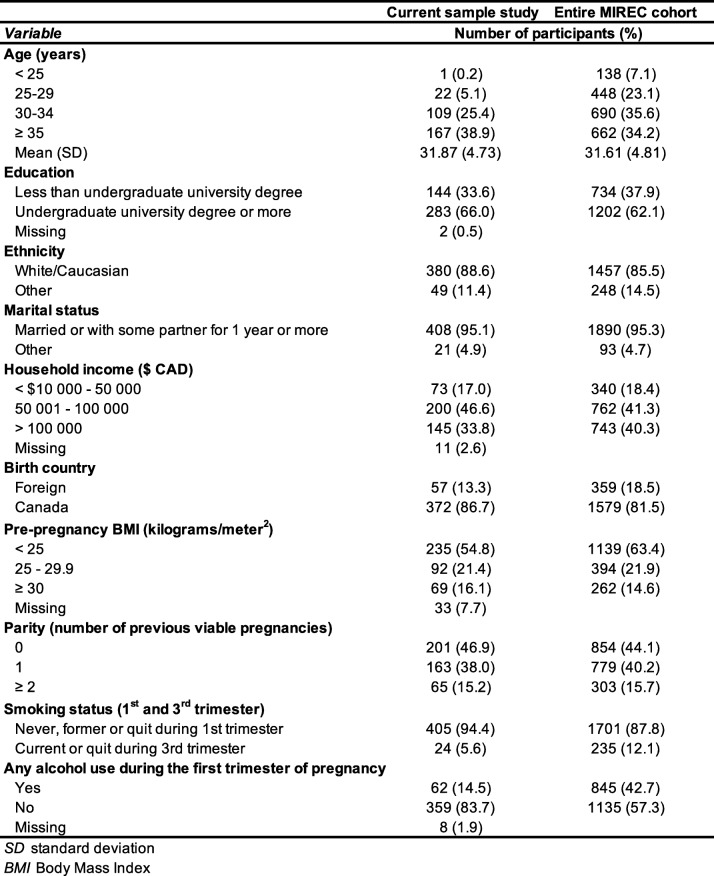
Material characteristics

Infant characteristics for the current study sample (*n* = 429) and for the entire MIREC cohort (*n* = 1983) are shown in Table 2. In the current study, fourteen (3.3%) babies were born moderately to late preterm (33 to < 37 weeks of gestation). Sixteen (3.7%) had a low birth weight (< 2500 g), of which seven were both premature and low birth weight. Infants were about 6 months of age at testing time (mean corrected age = 6.63, ranging from 4.1 to 9.8 months) and were mostly still breastfed (72.7%). Female and male infants were approximately equally represented. The data were almost identical than those of the entire MIREC cohort. At the testing time, the mean visual acuity scores, i.e., 5.66 cpd (*SD* = 2.97) and 8.98 cpd (*SD* = 2.50) for TAC and VEP respectively, where a higher cpd value indicates better visual acuity. These results are in the normal range for this age interval and higher VEP compared to TAC scores are to be expected [40]. Infants who only did TAC testing (*M* = 5.91; *SD* = 2.85 cpd) and those who did both TAC and VEP testing (*M* = 5.56; *SD* = 2.93 cpd), did not differ on TAC scores (*t*_(410)_ = 0.87, *p* = 0.38).

**Table 2:**
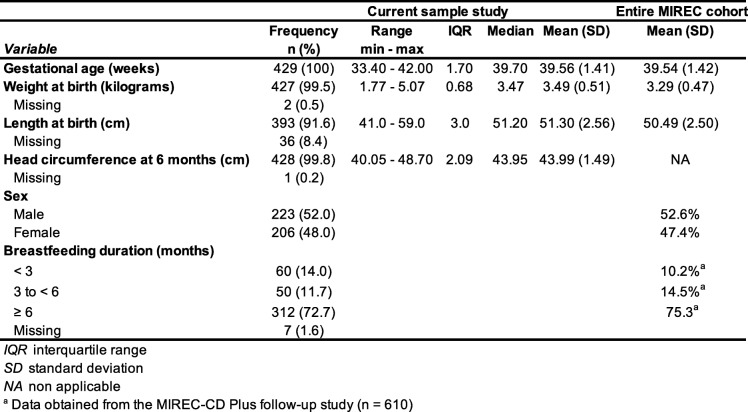
Infant characteristic

Descriptive statistics for environmental contaminants of interest are presented in Table 3.

**Table 3:**
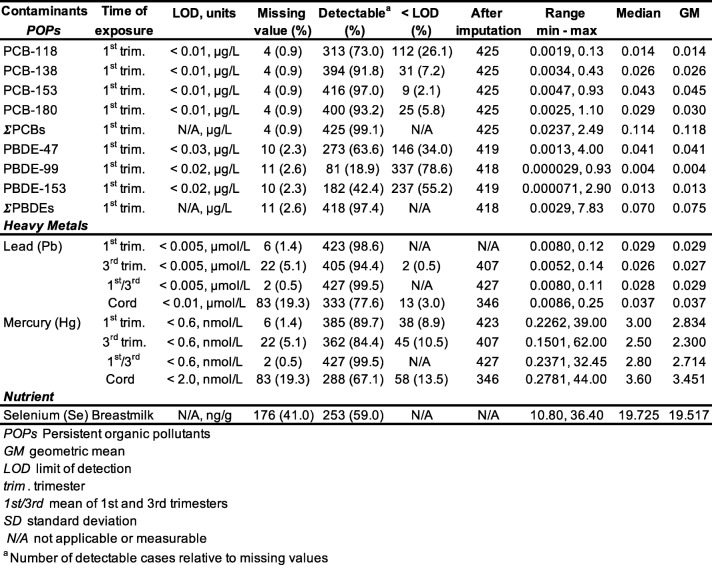
Descriptive statistics of contaminants and nutrients of interest (*n* = 429)

Blood samples taken during the 1st trimester of pregnancy showed that most of the women had detectable concentrations of PCBs, with congener 153 being, as expected, the most frequently detected (97.0% of the sample). PBDEs were detected less frequently for the same period (ranging from 18.9 to 63.6% depending on the congener). Heavy metals were detectable in all biological measures, cord blood showing higher mean values compared to mean 1st/3rd trimesters. Most of the women had detectable heavy metals in their blood (> 84%). POP levels in our sample were the same than those found in the entire MIREC cohort. For example, concentrations for PCB-153 were 0.043 and 0.043 μg/L respectively, and 0.041 vs. 0.042 μg/L for PBDE-47. Pb exposure in our sample was identical to the entire MIREC cohort (0.037 μmol/L), whereas Hg levels were slightly lower (3.6 vs. 3.99 nmo/L in the cord blood) [37]. Independent *t*-tests (results not shown) revealed no significant difference in ECs concentrations between the subsample of participants in the present study (*n* = 429), and those who participated in the MIREC-ID study but for whom no visual outcome was available (*n* = 96). Intercorrelations between contaminants of interest and selenium, as well as between different time points of exposure, are presented in Table 4. Correlations between contaminants are in the low to moderate range (*r’*s min.-max. = 0.01 to 0.38), whereas correlations between 1st and 3rd trimester exposure for Hg and Pb are high (*r’*s min-max. = 0.72 to 0.76).

**Table 4:**
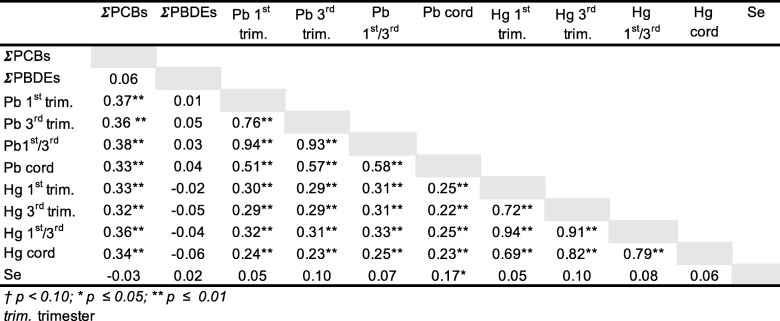
Intercorrelations among log-transformed concentration of contaminants and selenium

### Associations between ECs and behavioural visual acuity (TAC)

Table 5 show unadjusted and adjusted Beta coefficients for both types of analysis (categorically and continuously), for TAC visual acuity scores, for all contaminants of interest. Linear regression models using concentrations categorized in tertiles and in continuous log values revealed no association with ∑PCBs, before or after adjustment for covariates (all *p-values* > 0.05). Results were similar for ∑PBDEs, except for a marginal positive association in the third/highest exposure tertile (β for a 10-fold increase = 0.14, 95% CI = − 0.01, 0.29, *p* = 0.08). Considering the substantial number of imputed values for ∑PBDEs (particularly because of PBDE-99 and PBDE-153, see Table 3), regression modeling was also conducted only on PBDE-47, which was detected in most of the samples. No difference in the results were observed (data not shown), except for the marginal (positive) association between TAC and ΣPBDEs (see Table 5, Tertile 3) that disappeared (β for a 10-fold increase = 0.09, 95% CI = − 0.07, 0.24, *p* > 0.1). As seen in Table 5, no statistically significant associations between heavy metals and TAC were found for both exposure time points (mean of 1st/3rd trimesters or cord blood), for either type of regression analysis (categorical or continuous).

**Table 5:**
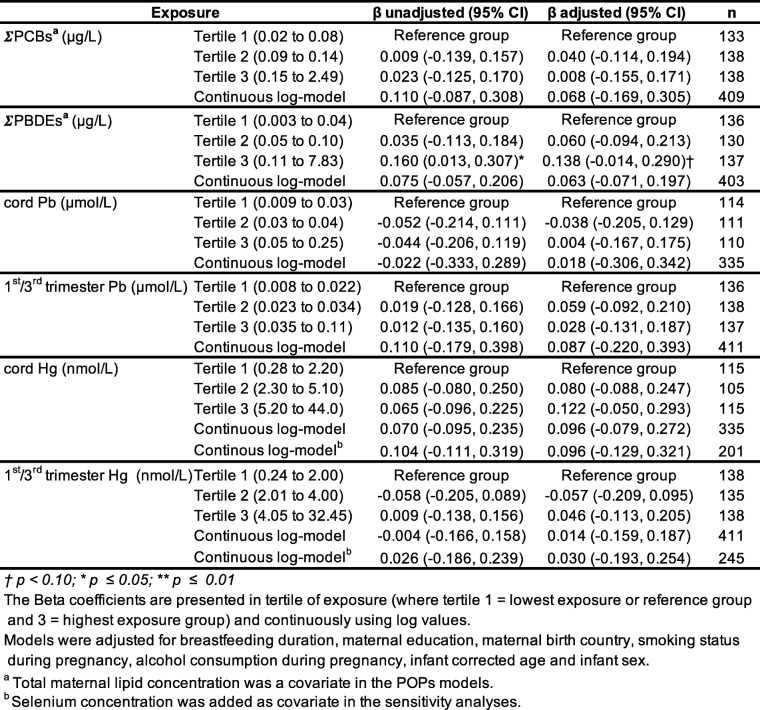
Association between contaminants and behavioral acuity scores

As a secondary aim, we tested for a potential modifier effect of sex in our data, as often reported in epidemiological studies including one from the MIREC cohort [49]. Thus, the data were stratified between boys and girls and the regression models were rerun (see Additional file 1: Table Supplement 1). Results were exactly the same for girls. For boys, the same patterns of results were observed, except for a significant association between TAC score and Hg that was not present in the main analysis (Table 5). However, this association was not robust, as it was positive for cord Hg levels, but negative for the mean 1st/3rd trimester’s Hg levels.

Given the potential effect of selenium against Hg neurotoxicity reported in the literature [50], further analyses were conducted to specifically explore the potential influence of selenium on the Hg associations. First, breast milk selenium was added as a covariate to the Hg regression models. As shown in Table 5, the associations between Hg (cord as well as mean 1st/3rd trimester exposures) on TAC scores did not change their direction, nor their strength after adjusting for selenium concentration. To further the potential influence of selenium, we analyzed the regression models by stratifying the selenium distribution, although selenium concentration was available only for about half of the sample (*n* = 231) (Table 6). Thus, two groups were created based on the median value of selenium concentration, i.e., the low-exposed group (< 19.80 ng/g) and the high-exposed group (≥ 19.80 ng/g). This cut-off value corresponds to the recommended Adequate Intake (AI) dietary reference for selenium among 0–12 months infants (i.e., between 15 and 20 μg/day) [51]. The analyses did not reveal any difference in the patterns of results for TAC scores in association to Hg exposure (see results in Table 6).

**Table 6:**
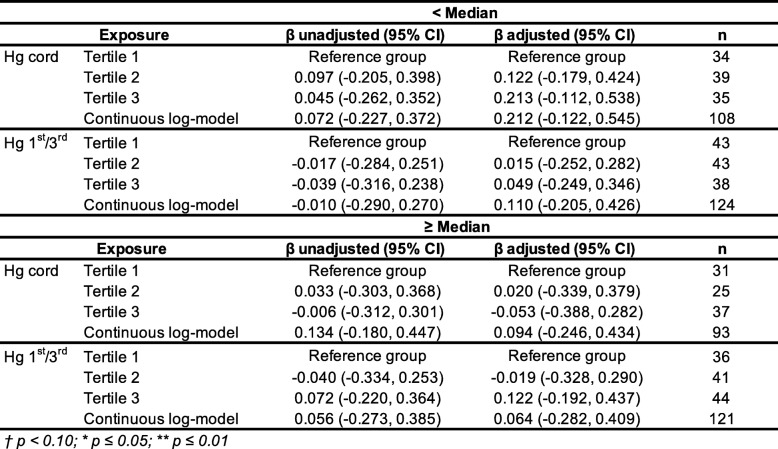
Stratified analysis is for selenium concentration at median (19.80 ng/g) for the TAC model

Sensitivity analyses were also conducted to confirm our initial results. First, we reran the analysis for the TAC model excluding participants considered outliers because of extremely low behavioural acuity scores (< 1st percentile) based on the test norms (*n* = 17). The same results were obtained, except for the positive association between PBDEs and TAC that was no longer apparent when excluding outlier participants (see Additional file 1: Table Supplement 2). Of note, these 17 infants did not statistically differ from the others (*n* = 429) in terms of age (*t* (423) = − 0.56, *p* = 0.58) or exposure concentrations for ∑PBDEs (*t* (419) = 0.94, *p* = 0.35), ∑PCBs (*t* (426) = − 0.04, *p* = 0.97), cord Hg (*t* (347) = − 1.71, *p* = 0.09), mean trimesters Hg (*t* (428) = − 1.84, *p* = 0.09), cord Pb (*t* (347) = − 0.74, *p* = 0.46) or mean trimesters Pb (*t* (428) = − 0.44, *p* = 0.66). The second sensitivity analysis excluded premature and/or low birth weight infants (*n* = 23) from the sample size. Once again, the results remain unchanged from the initial analysis, except for the positive association between PBDEs and TAC that was no longer observed (see Additional file 1: Table Supplement 3). Third, given the relatively low inter-correlations between PCBs, PBDEs, Hg and Pb, a single model was calculated with simultaneous adjustments for all exposures. The pattern of results did not changed (see Additional file 1: Table Supplement 4). Finally, a fourth sensitivity analysis was conducted by adjusting the associations in the continuous models for site, as the participants of the current study were recruited and followed from 7 Canadian centers. Null associations were still observed, as illustrated here for the main variables of interest, i.e., 0.077 (− 0.154, 0.308) for ΣPCBs, 0.081 (− 0.048, 0.209) for ΣPBDEs, 0.129 (− 0.188, 0.447) for cord Pb, and 0.133 (− 0.042, 0.307) for cord Hg.

### Associations between ECs and electrophysiological (VEP) visual acuity

Table 7 show unadjusted and adjusted Beta coefficients for both types of analysis (categorically and continuously), for VEP visual acuity scores, for all contaminants of interest. Descriptive statistics for environmental contaminants of interest for the VEP subsample (*n* = 63) are presented in Additional file 1: Table Supplement 5). All linear regression models using contaminant concentrations categorized in two groups (≤ and > median) based on the sample size revealed no association with VEP acuity scores. As for continuous log value analyses, they also showed no clear pattern of association between VEP scores and POPs (∑PCBs, ∑PBDEs), as well as heavy metals (Pb and Hg) when using the 1st/3rd trimester concentrations. However, cord blood Pb (β for a 10-fold increase = − 2.99, 95% CI = − 6.39, 0.40) and cord blood Hg (β for a 10-fold increase = − 1.90, 95% CI = − 4.14, 0.34), were marginally associated with a decrease of VEP visual acuity. We investigated the potential influence of breastmilk selenium concentrations on the Hg models. These analyses were considered exploratory as selenium measurement was available for about half of the sample. Result showed that the initial marginal association between Hg and decreased VEP visual acuity in the continuous log model (Table 7) became statistically significant when selenium was added as a covariate (β for a 10-fold increase in cord blood Hg = − 3.41, 95% CI = − 5.96, − 0.86). A similar significant decrease of VEP acuity was also revealed for the mean 1st/3rd trimester’s Hg exposure (β for a 10-fold increase in mean trimesters Hg = − 3.29, 95% CI = − 5.69, − 0.88), which was not apparent before adjustment for selenium levels (Table 7). To determine whether this effect was due to an adjustment of Se or to a change in sample size, we re-conducted the Hg models while including only participants with selenium data (i.e., *n* = 33 instead of 63). Results revealed that the significant associations between Hg and VEP acuity became much stronger. However, they did not change when adjusting for selenium (Additional file 1: Table Supplement 6).

**Table 7:**
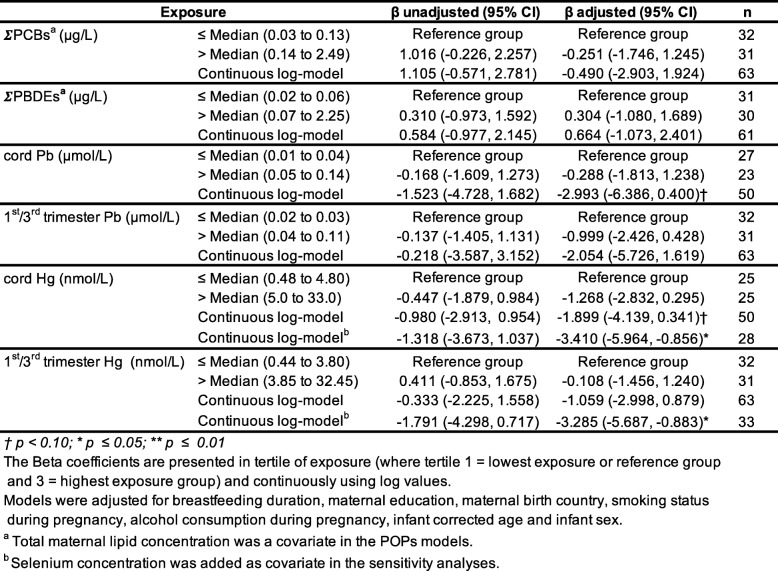
Associations between contaminants and electrophysiological acuity scores

## Discussion

This study aimed to assess the potential neurotoxic impact of prenatal exposure of two classes of legacy ECs (POPs and heavy metals) on visual acuity, among 6-month-old healthy infants from the Canadian general population. No statistically significant association between prenatal exposure to PCBs or PBDEs and any of the visual acuity outcomes (TAC or VEP) was observed. Although prenatal exposure to heavy metals was unrelated to TAC scores, cord Pb and cord Hg were associated with decreased VEP acuity. Exploratory analyses on the potential influence of selenium exposure on Hg association revealed stronger and statistically significant results when selenium was entered in the regression models, either for cord blood Hg or mean 1st/3rd trimesters Hg concentrations. This apparent selenium-based modifier effect was, however, not confirmed when the number of participants in the two models, i.e., with or without selenium concentrations, was controlled. This suggests that the estimation change in the main analysis might not due to adjustment for selenium, but instead to a change in the study sample. However, this interpretation needs to be taken with cautious since the sample sizes in all of these analyses were very small to ensure robust and valid regression modeling.

### Associations between POP exposure and visual function

When we looked at the sample as a whole (*n* = 429), the highest tertile of ΣPBDEs concentration (0.1 to 7.83 μg/L) was marginally associated with increased behavioural (TAC) visual acuity. This result was somewhat unexpected given the low level of exposure in this study cohort and the positive direction of the association. However, sensitivity analyses (Additional file 1: Table Supplement 2 and 3) on participants without extreme scores or who were premature/low birth weight revealed that this ΣPBDEs/TAC association was no longer detectable, suggesting that the initial association was likely created by the influence of outlier scores on the fit of the regression line. That being said, it is not uncommon to obtain positive associations between exposure and neurodevelopmental outcomes in epidemiological studies, mostly when examining the impact of very low exposure, as found in the general population. For example, in the Dutch COMPARE study cohort, prenatal exposure to POPs (PBDEs and PCBs) has been associated both positively and negatively to neurodevelopmental outcomes at age 5–6 years [52].

In regard to the visual domain, there is very limited evidence of deficits in relation to prenatal POPs exposure. To the best of our knowledge, none of the POPs of interest (PCBs or PBDEs) have been previously examined in regard to visual acuity. The only data available in the literature come from a few VEP studies investigating prenatal exposure to PCBs in relation to visual processing, i.e., the cortical responses evoked by a visual stimulus. These studies, assessing a global visual outcome rather than acuity which is more specific, reported no significant association [15, 16, 53, 54]. Moreover, in line with our findings, no significant association between prenatal exposure to chlordecone, a POP pesticide that was intensively used in the French West Indies, and TAC visual acuity was observed among 7-month-old Guadeloupean infants [55].

In addition to prenatal exposure, postnatal exposure to POPs has been studied in association with visual functions. It is important to point out, however, that because of the bioaccumulative properties of the POPs compounds and their long half-life, early postnatal exposure is not totally independent of prenatal exposure. Some VEP studies have shown subtle deficits associated with postnatal exposure to POPs. For instance, background perinatal levels of various POPs (i.e., PCBs, DDE, DDT) in colostral milk have been found to be associated with VEP alterations at 12 months of age [56]. Furthermore, subtle VEP delays were observed in association with PCB concentrations measured at 5 years of age among Inuit children from Arctic Québec (Canada) [16]. However, PCB concentrations in this latter study were more than 10-fold higher than in the present cohort. Finally, among adults, only one study reported a statistically significant impact of POPs exposure on vision (i.e., reduced colour discrimination, visual field constriction) in association with long-term and high exposure to PCBs throughout adulthood [57].

It has been previously suggested that each class of ECs could have their own particular sphere of brain alterations or deficits (e.g., sensory vs. cognitive) [58]. For instance, there is evidence that PCBs might predominantly affect the cognitive domain (e.g., executive functions, visuospatial abilities, attention), whereas alterations of sensory functions are more commonly associated with heavy metals [52, 59]. In agreement with this notion, we did not observe any significant association between POPs and visual outcomes (but see below for lead and mercury). In fact, there is increasing literature showing cognitive and behavioural alterations in relation to very low-level POP exposure, either for PCBs or PBDEs [60, 61]. For instance, a recent MIREC behavioural study in infants reported that prenatal PBDEs were associated with a higher propensity to frustration at age 7 months, as measured with the arm restraint task [62].

### Associations between heavy metal exposure and visual function

In contrast to POPs, the vulnerability of the visual system to heavy metal exposure is well known. For instance, occupational exposure to Pb or Hg can disrupt specific visual functions (i.e., colour discrimination, contrast sensitivity, visual acuity) [63, 64], as well as some physiologic aspects of the eye (i.e., visual field constriction, lenticular changes) [65, 66]. In children, VEP studies have reported significant associations between prenatal [13–16] and postnatal [16, 67] chronic exposure to heavy metals and visual processing deficits.

Few studies have looked at specific visual functions such as visual acuity or contrast sensitivity, and the current data are equivocal. For example, deficits in contrast sensitivity, but not visual acuity, have been found among Bohemian children in the Czech Republic exposed to ambient levels of Hg from a polluted area [68]. In the Faroe Islands, prenatal exposure to Hg has not been clearly associated with contrast sensitivity deficits among 7-year-old children [69]. Even fewer studies are available regarding exposures at current low levels. In one cohort study of 6-year-old children within the general German population exposed to heavy metals (*n* = 384), Altmann et al. [12] reported no associations with Hg exposure, but suggested associations between postnatal Pb and some of the VEP latencies, suggesting prolonged neural time conduction and/or reduced intracortical activity. These results were, however, marginal, as only 3 VEP outcomes over 21 were found to be statistically significant. In the same study, the assessment of psychophysical contrast sensitivity was done (i.e., where stimuli are presented at various levels of contrast, until a threshold is reached at which the subject can no longer judge the stimulus as perceptible). The results showed impaired contrast sensitivity scores with increasing postnatal Hg exposure for some spatial frequencies but in a subtle and an inconsistent way (i.e., for 1.5 and 3 cpd in the right eye, and 3 and 18 cpd in the left eye). No statistically significant associations were found for Pb. Although these results suggest the alterations of visual functions in associations to Hg and Pb might be modest, visual acuity and contrast sensitivity in particular are nevertheless important variables to take into account in multiple regression models when assessing cognitive outcomes (i.e., in visual sustained attention task) [70].

To our knowledge, only one cohort study has investigated infant sensory function in relation to background-level Pb exposure [25]. Prenatal Pb exposure was measured in a large study (*n* = 1019) in rural northeastern China at various time points, i.e., during middle (≈15.5 weeks), late pregnancy (≈39 weeks), and at delivery (cord blood). Regression analyses where exposure was categorized in tertiles for maternal whole blood (< 2, 2–3.8, > 3.8 μg/dL) and cord blood (< 2, 2–3.2, > 3.2 μg/dL) showed that TAC scores among neonates were lower in association with higher late-pregnancy Pb concentrations. Compared to the TAC scores of infants born from mothers who had low late-pregnancy Pb, those whose mothers had higher late-pregnancy Pb (i.e., 2–3.8 and > 3.8 μg/dL) had respectively mean TAC scores that were 7.2 and 8.5% lower. There was no significant association between TAC scores and the other time points of Pb measurement (i.e., mid-pregnancy or cord blood). In the current study, we also examined exposures at different time points during pregnancy, i.e., 1st and 3rd trimesters and at birth (cord blood). Average Pb concentrations during the 3rd trimester was 0.032 μmol/L (i.e., 0.66 μg/dL) and the most highly exposed group (third tertile) for mean 1st/3rd trimester exposure was subject to concentrations equivalent to 0.035 to 0.11 μmol/L (i.e., 0.72 to 2.28 μg/dL). Therefore, the highest Pb exposure group in our sample is approximately equivalent to the lowest Pb exposure group of the Chinese study (i.e., < 2 μg/dL). We did not find any significant association between exposure during pregnancy and TAC scores. However, we did find a negative association (*p* < 0.1) between VEP visual acuity scores and Pb cord blood where for a 10-fold increase in cord Pb exposure, there was a decrease of 3 cpd in VEP acuity, which is clinically significant, i.e., corresponds to a change of at least one line in the Snellen visual chart commonly used by eye care professionals. Interestingly, a reduction of about the same magnitude in VEP acuity scores (2.94 cpd) has been reported in children exposed prenatally to solvents [71].

In the Chinese cohort study [25], Pb concentrations were higher for maternal blood during pregnancy compared to cord blood. In the present MIREC cohort study, however, cord blood levels were slightly higher compared to maternal blood, as reported elsewhere [72, 73]. The biological distribution of Pb during pregnancy is not well understood, and a U-shaped pattern over the trimesters has been proposed, at least in populations with relatively high levels (i.e. ≥ 2 μg/dL) [37]. Many variables can possibly modify Pb exposure during pregnancy, such as calcium or iron intake, smoking, maternal age and socioeconomic status [74]. Although we might think that the time window with the highest levels of Pb during the pregnancy is the most powerful to predict infant neurodevelopment, this is not necessarily the case. For instance, infants chronically exposed to prenatal Pb from the Mexico City cohort showed a significant reduction in the Bailey mental development index in association with the 1st trimester exposure, but not the 2nd or the 3rd, although the levels between the 3 time periods were quite similar [75]. In another study, exposure during the 3rd trimester (28–36 weeks) was found to be the most sensitive period to predict school-age child intellectual development, even though the blood Pb levels of the second trimester were slightly higher [76]. This reinforces the concept of critical developmental window and the knowledge that a toxic insult can cause more persistent and irreversible damage if it occurs during an exponential development phase [77].

In contrast to Pb, the most important associations with visual acuity in the present study were observed for Hg exposure, which has not previously been assessed by any other cohort study as far as general population and exposure at background levels are concerned. We found that the initial association between VEP scores and cord Hg exposure became statistically significant after adjustment for breast milk selenium (Se) concentration, so that a 10-fold increase in Hg concentration was associated with a 3.4 cpd decrease of acuity. Also, a significant association emerged between mean 1st/3rd trimester Hg exposure and VEP, again after adjustment for Se. These results are consistent with other epidemiological studies that did not find significant associations between Hg exposure and neurodevelopmental outcome before adjusting for selenium in their regression models [13, 53]. Selenium is an essential trace mineral that is known for its antioxidant properties. Several animal studies have demonstrated that Se intake may alter MeHg toxicity, reducing reproductive and developmental alterations [78]. Even though clear evidence in epidemiological studies is lacking, some have proposed that selenium may have neuroprotective effects against human Hg toxicity [79]. The mechanisms underlying the protective effect of selenium on Hg are complex and the physiologic functions of Se in human brain are not well understood, probably involving protection against oxidative stress and regulation of neuronal and thyroid function and metabolism [80]. Using stratified analysis based on the median value of Se in breast milk (< or ≥ 19.80 ng/g), we did not find significant differences in the relationship between Hg and TAC scores. This is not surprising considering that the great majority of our participants reached the recommended intake of Se based on the Adequate Daily Intake for this particular age group (i.e., between 15 and 20 μg/day). Also, 87% of the sample presented a Se value of > 15 ng/g, which is coherent to the average concentration of Se in breastmilk measured in North America (i.e., 15 to 20 μg/L) [38]. Therefore, our stratified analysis approach was probably lacking sensitivity to capture group differences since the Se levels in the low-level group were actually not that low. Also, the important time difference between the measurement of Se (in breast milk between the 2nd and the 10th postnatal weeks) and prenatal Hg (during pregnancy and at delivery) constitutes another limitation of this stratified analysis, although it has been reported that maternal blood selenium (before delivery), cord blood and maternal milk are strongly correlated (*r*_s_ > 0.6) [39].

### Visual acuity as a marker of subtle neurotoxicity to low-level ECs

Several molecular and cellular processes have been implicated in the neurotoxicity of both Hg and Pb, such as impairments in neural differentiation, synaptogenesis and myelination [81]. It can be hypothesized that the subtle reduced visual acuity observed in our study in relation to prenatal heavy metals exposure might be linked to a delay or alteration of the myelination of the visual tract, which has also been proposed in studies that found slower processing speed using VEP [15, 16]. Moreover, the visual acuity function has a large critical period of development, from the embryogenic period to school age [82], which suggests that potential alterations might originate from both prenatal and/or early postnatal periods. There is increasing evidence about delayed neurotoxicity in humans, principally for Hg exposure, which is clearly established in animal models [83]. Thus an early insult to the visual system can have long-term adverse consequences on some maturational processes (e.g., synaptic remodeling and pruning) [84], which might lead to functional alterations on later neurodevelopment. This notion implies that behavioural visual acuity can be in the normal range at 6 months of age, as observed in the present study with the TAC, but can decrease later in life in association with prenatal exposure.

### Comparison of exposure levels with other general population cohort studies

Prenatal heavy metals exposure in this study were among the lowest of all other general population cohort studies carried out around the globe, as for example, in Korea [85], the UK [86] or Spain [87]. This result is in accordance with the study of Foster et al. [88], which showed lower levels of Pb and Hg in pregnant Canadian women, compared to those reported in the other international studies. Although our Canadian maternal blood levels were low, they are in the same range as those reported in some U.S. general population cohorts [89, 90].

In our sample, levels of PCBs were also lower than those measured in the U.S. [91], and up to 5 to 9-fold lower than European levels [92, 93]. For PBDEs, maternal levels were also 4 to 5 times lower than the ones found in the U.S. [94, 95], but higher than the ones found in Europe [52, 96], which is in accordance with higher exposure in North America compared to Europe or Asia. PBDE-153 exposure in our sample was similar to two other cohort studies carried in Canada [88, 97].

Despite the low levels of environmental contaminants measured in our sample, maternal mercury exposure was significantly associated with lower electrophysiological visual acuity score, when selenium was added to the regression model. The results found in the present study are in accordance with the recent body of literature suggesting subtle but measurable negative associations between neurodevelopmental outcomes and low-level exposure in the general population.

### Strengths and limitations

To our knowledge, this is the first study to examine background low-levels of ECs in relation to visual acuity development in Canada. This study has a number of strengths. Regarding heavy metals exposure, three prenatal time points were measured during the pregnancy, which allows a certain temporal specificity when looking at potential neurotoxic effects. Lifestyle, anthropometric and demographic questionnaires, which were administered multiple times during pregnancy and at delivery were also very detailed, providing precise and repeated covariate data. This study, however, is limited by the fact that for POPs exposure, only measurable data for the 1st trimester exposure was available, compared to the addition of the 3rd trimester and cord blood for heavy metals, which might have decreased the possibility of finding effects on visual acuity development. Furthermore, regression models for ∑PBDEs were based on a substantial number of imputed values for ∑PBDEs, i.e., on values under the LOD, which might has affected the power and accuracy of the estimates. Also, we did not investigate potential additive or synergic effects between ECs, or with other unmeasured chemicals present in the environment such as air pollution particles. Selenium in breast milk was considered as an indirect indication of maternal status during pregnancy. However, some limitations arise from the distance in time between both measurements. From a statistical point of view, only deleterious effects on VEP acuity have been found. However, our sample size for the latter analysis was very limited to ensure robust and valid regression modeling, and even more so when selenium was added to the model. Finally, mothers in our sample were on average of a higher socio-economic class than the general population of women giving birth in Canada at the same time [29], which can explain some of the lower levels of exposure and the possible interaction of protective factors in the effect of ECs on visual development, such as a stimulating home environment and good nutrition. This subsample represents a specific upper class that might exhibit lower risk in terms of neurotoxic effects as compared to the overall Canadian population and therefore, our results cannot be generalized to the entire Canadian population. Future studies should aim to replicate these findings in a larger sample and in other countries.

## Conclusions

In this study, visual acuity among 6-month-old infants was assessed using two approaches: the behavioural (TAC) and electrophysiological methods (VEP). Using TAC, no alteration in the visual acuity function was detected. However, using VEP, we found subtle alterations in visual acuity function measured in association with low levels of prenatal heavy metals, whereas no association was found with prenatal POPs exposure. These results are consistent with the emerging literature supporting the idea that even at background/low levels, exposure to some ECs can have subclinical but measurable impact on child development. Our results also suggest that the VEP assessment might be more sensitive to detect subclinical alterations to developmental exposure. It is noteworthy that TAC is a behavioural test that requires visuomotor integration and oculomotor responses [98]. Thus, both tests assess visual acuity function but not by measuring the same brain structures; whereas the TAC reflects the integrity of a relatively large visual brain network, the VEP are more specific to the visual cortex activity. Subtle visual alterations during infancy may cause long-term consequences on cognition and learning at school age. Our results suggest that more epidemiological studies should assess visual function development in relation to prenatal EC exposure, not only as a covariate but as a variable of interest.

## Supplementary information

**Additional file 1**: Table Supplement 1. Stratified analysis using infant’s sex (boys and girls) for the TAC model (*n* = 429). Table Supplement 2. Association between contaminants and behavioral acuity scores (TAC) excluding outliers (*n* = 412). Table Supplement 3. Association between contaminants and behavioral acuity scores (TAC) without low birth weight and premature babies (*n* = 389). Table Supplement 4. Association between contaminants and behavioral acuity scores (TAC) with simultaneous adjustment for all exposures. Table Supplement 5. Descriptive statistics of contaminants and nutrients of interest for the VEP sample (*n* = 63). Table Supplement 6. Association between Hg and VEP acuity using only participants with selenium concentration.

## Data Availability

Access to the data is on a cost-recovery basic through the MIREC Biobank processes (www.mirec-canada.ca). The MIREC Biobank policy does not allow the transfer outside Canada of individual level data.

## Abbreviations

Cpd: Cycle per degree of visual angle
EC: Environmental contaminants
Hg: Mercury
LOD: Limit of detection
MIREC: Maternal-Infant Research on Environmental Chemicals Study
Pb: Lead
PBDE: Polybrominated diphenyl ethers
PCB: Polychlorinated biphenyls
POP: Persistent organic pollutants
Se: Selenium
TAC: Teller acuity cards
VEP: Visual evoked potentials
