# Supplementary material for: Prenatal exposure to legacy contaminants and visual acuity in Canadian infants: a maternal-infant research on environmental chemicals study (MIREC-ID)
Source: Environ Health. 2020 Feb 7;19:14. doi: 10.1186/s12940-020-0567-2 (PMC7006412; doi:10.1186/s12940-020-0567-2)
Supplement: Supplementary file 1 — Additional file 1: Table Supplement 1. Stratified analysis using infant’s sex (boys and girls) for the TAC model (n = 429). Table Supplement 2. Association between contaminants and behavioral acuity scores (TAC) excluding outliers (n = 412). Table Supplement 3. Association between contaminants and behavioral acuity scores (TAC) without low birth weight and premature babies (n = 389). Table Supplement 4. Association between contaminants and behavioral acuity scores (TAC) with simultaneous adjustment for all exposures. Table Supplement 5. Descriptive statistics of contaminants and nutrients of interest for the VEP sample (n = 63). Table Supplement 6. Association between Hg and VEP acuity using only participants with selenium concentration. [file 12940_2020_567_MOESM1_ESM.docx]

Supplement materials
